# Supplementary material for: TP53INP2 Promotes Bovine Adipocytes Differentiation Through Autophagy Activation
Source: Animals (Basel). 2019 Dec 2;9(12):1060. doi: 10.3390/ani9121060 (PMC6940805; doi:10.3390/ani9121060)
Supplement: Supplementary file 1 [file animals-09-01060-s001.pdf]

Supplementary file

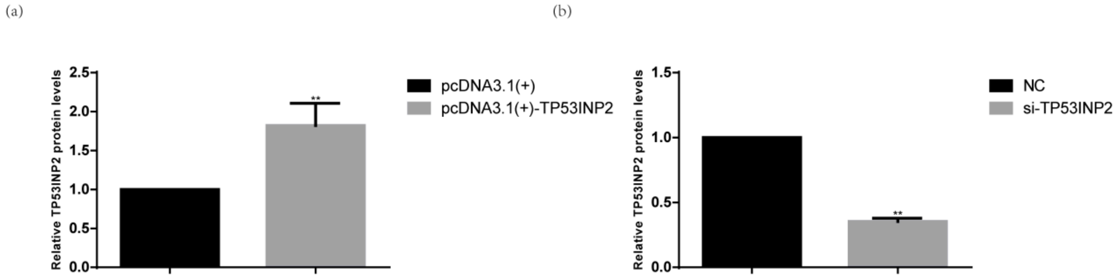

Figure 1. Protein quantitative analysis of overexpressing and interference efficiency. (**a**) The quantification of protein levels after overexpressing of TP53INP2. (**b**) The quantification of protein levels after interfering of TP53INP2.

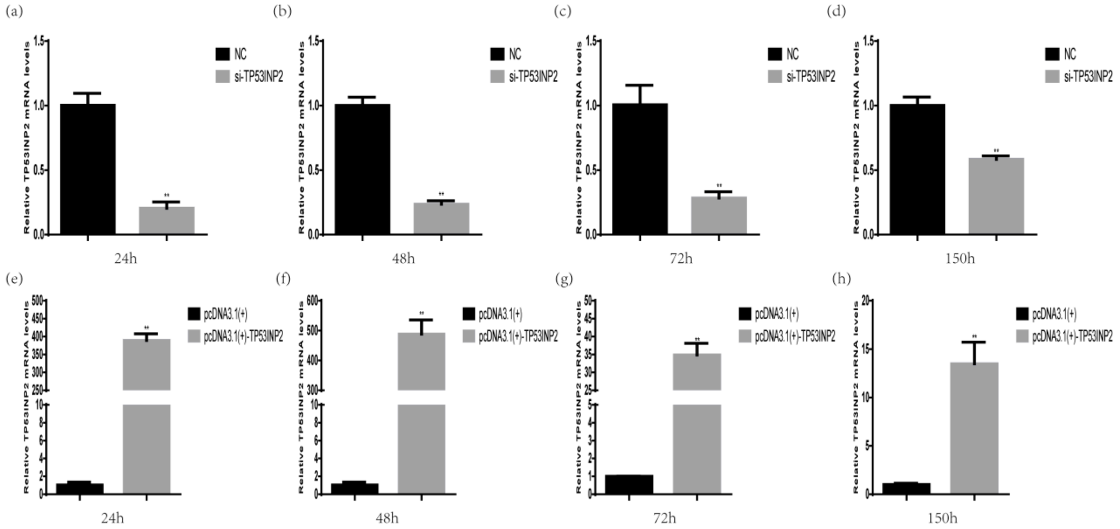

Figure 2. Detection of overexpression and interference efficiency of mRNA levels in different stages. Overexpression efficiency of mRNA levels at (**a**) 24 h, (**b**) 48 h, (**c**) 72h and (**d**) 150h. Interference efficiency of mRNA levels at (**e**) 24 h, (**f**) 48 h, (**g**) 72h and (**h**) 150h.

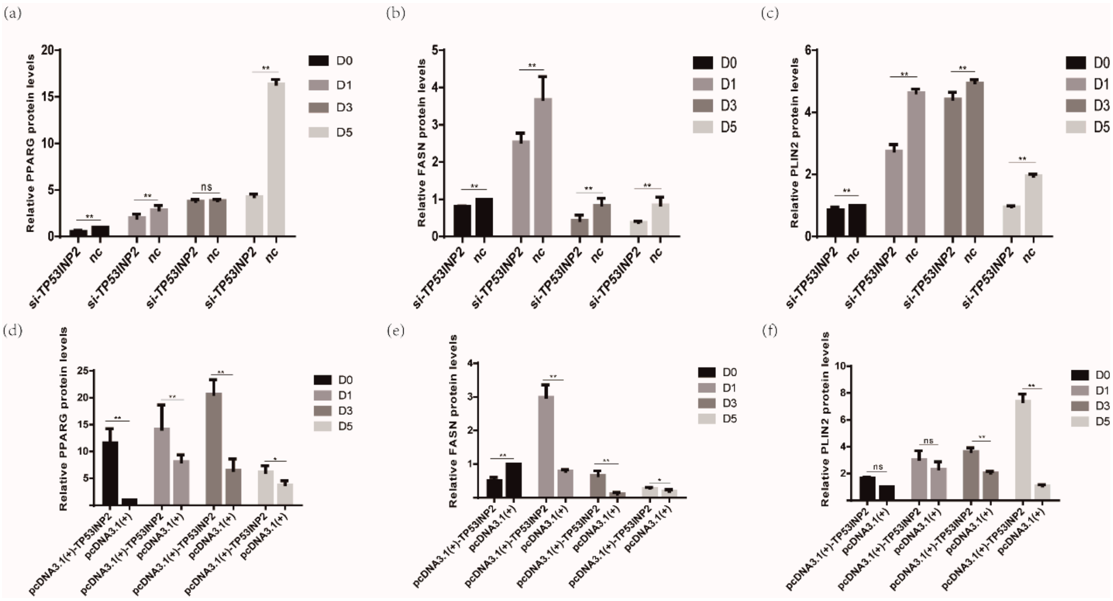

Figure 3. Quantified the adipocytes differentiation-related protein levels. Protein quantitative analysis of (**a**) PPARγ, (**b**) FASN and (**c**) PLIN2 after interfering of TP53INP2. Protein quantitative analysis of (**d**) PPARγ, (**e**) FASN and (**f**) PLIN2 after overexpressing of TP53INP2.

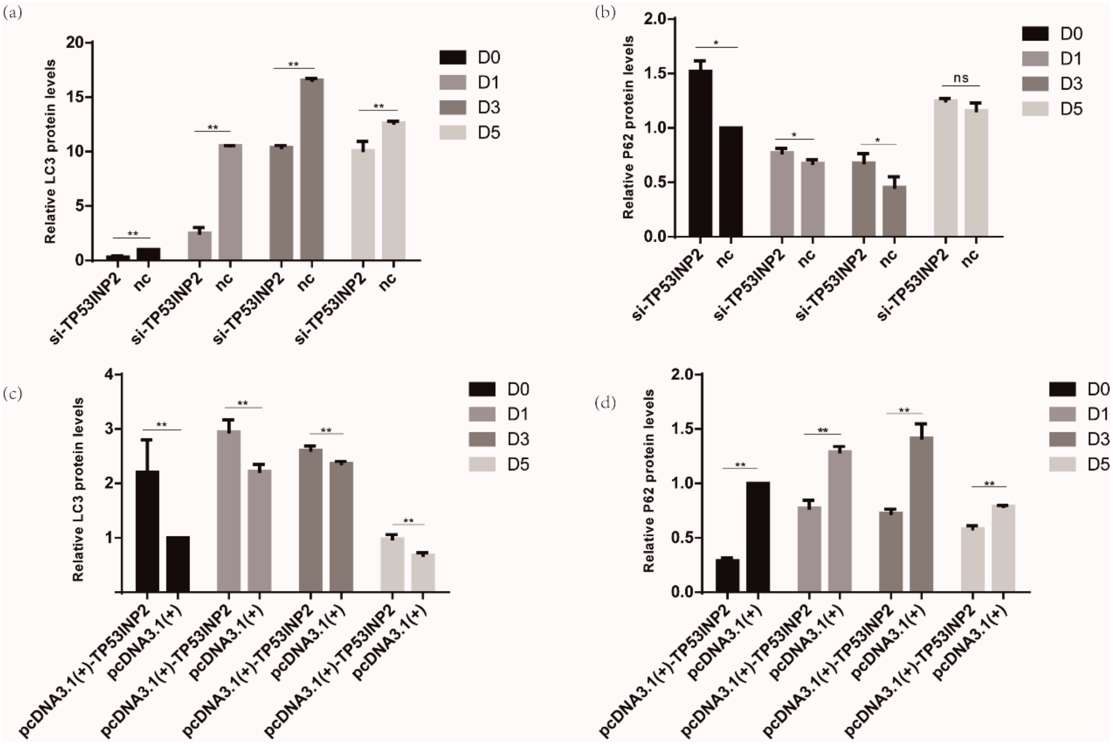

Figure 4. Quantified the autophagy-related protein levels. Quantified the protein levels. Protein quantitative analysis of (**a**) LC3, (**b**) p62 after interfering of TP53INP2. Protein quantitative analysis of (**d**) LC3, (**e**) p62 after overexpressing of TP53INP2.

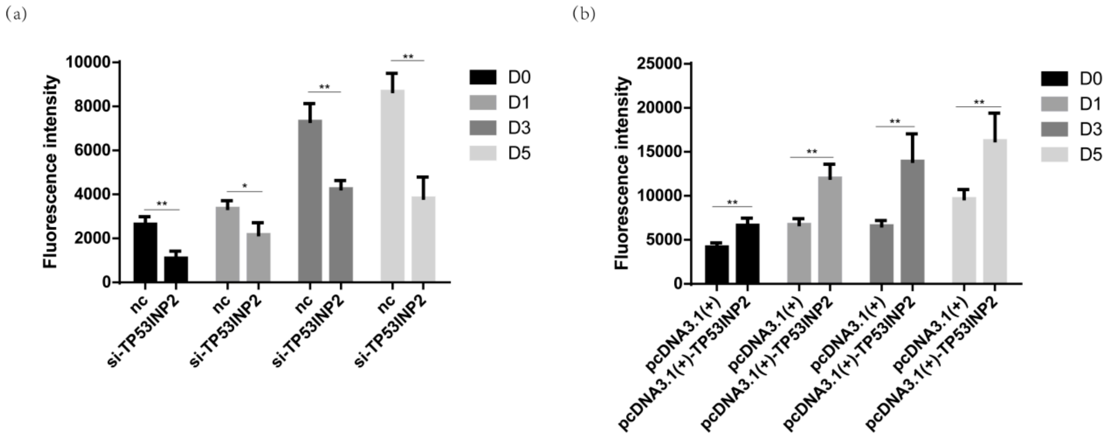

Figure 5. Quantification of the LipidTOX™ stain images. (**a**) Images quantitative analysis of LipidTOX™ stain after interfering of TP53INP2. (**b**) Images quantitative analysis of LipidTOX™ stain after overexpressing of TP53INP2.

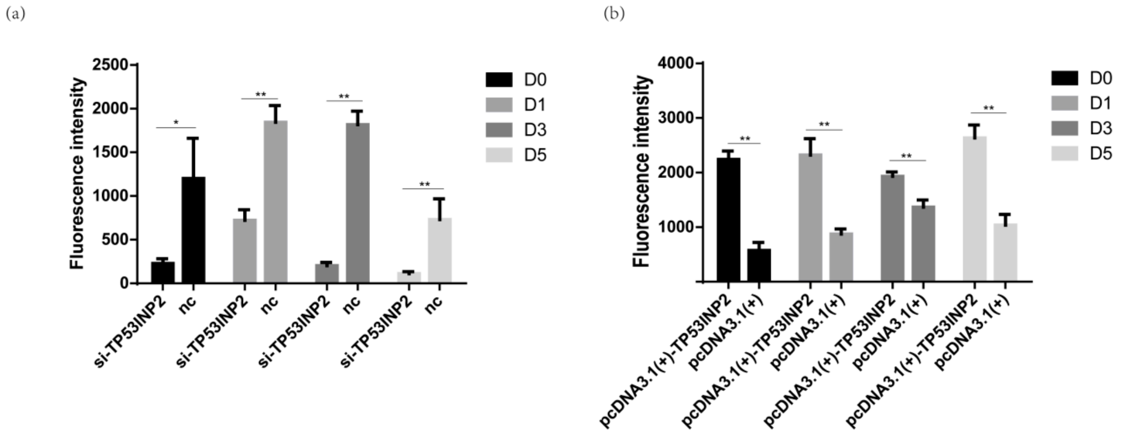

Figure 6. Quantification of the MDC stain images. (**a**) Images quantitative analysis of MDC stain after interfering of TP53INP2. (**b**) Images quantitative analysis of MDC stain after overexpressing of TP53INP2.

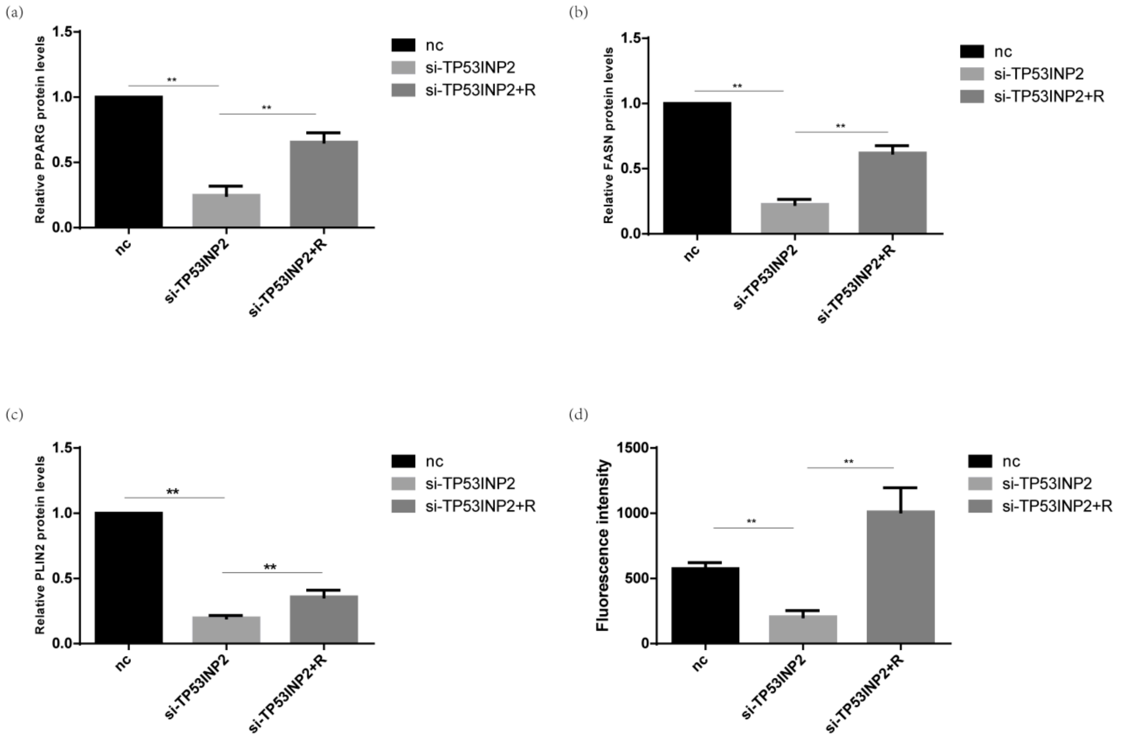

Figure 7. Quantification of the rescue experiment results. Protein quantitative analysis of (**a**) PPARγ, (**b**) FASN and (**c**) PLIN2. (**d**) Images quantitative analysis of LipidTOX™ stain.
